# APOBEC3A Is a Specific Inhibitor of the Early Phases of HIV-1 Infection in Myeloid Cells

**DOI:** 10.1371/journal.ppat.1002221

**Published:** 2011-09-22

**Authors:** Gregory Berger, Stéphanie Durand, Guillaume Fargier, Xuan-Nhi Nguyen, Stéphanie Cordeil, Serge Bouaziz, Delphine Muriaux, Jean-Luc Darlix, Andrea Cimarelli

**Affiliations:** 1 Department of Human Virology, ENS-L, Lyon, France; 2 INSERM, U758, Lyon, France; 3 University of Lyon, Lyon I, IFR128, Lyon, France; 4 UMR 8015 CNRS, University Paris Descartes, Paris, France; Fred Hutchinson Cancer Research Center, United States of America

## Abstract

Myeloid cells play numerous roles in HIV-1 pathogenesis serving as a vehicle for viral spread and as a viral reservoir. Yet, cells of this lineage generally resist HIV-1 infection when compared to cells of other lineages, a phenomenon particularly acute during the early phases of infection. Here, we explore the role of APOBEC3A on these steps. APOBEC3A is a member of the APOBEC3 family that is highly expressed in myeloid cells, but so far lacks a known antiviral effect against retroviruses. Using ectopic expression of APOBEC3A in established cell lines and specific silencing in primary macrophages and dendritic cells, we demonstrate that the pool of APOBEC3A in target cells inhibits the early phases of HIV-1 infection and the spread of replication-competent R5-tropic HIV-1, specifically in cells of myeloid origins. In these cells, APOBEC3A affects the amount of vDNA synthesized over the course of infection. The susceptibility to the antiviral effect of APOBEC3A is conserved among primate lentiviruses, although the viral protein Vpx coded by members of the SIV_SM_/HIV-2 lineage provides partial protection from APOBEC3A during infection. Our results indicate that APOBEC3A is a previously unrecognized antiviral factor that targets primate lentiviruses specifically in myeloid cells and that acts during the early phases of infection directly in target cells. The findings presented here open up new venues on the role of APOBEC3A during HIV infection and pathogenesis, on the role of the cellular context in the regulation of the antiviral activities of members of the APOBEC3 family and more generally on the natural functions of APOBEC3A.

## Author Summary

Macrophages and dendritic cells represent important targets for HIV-1 and the understanding of the complex relationship established between these cells and the virus is of the outmost importance. Here, we show that APOBEC3A, the least known member of the APOBEC3 family of cytidine deaminases, restricts HIV-1 specifically in these cells. The antiviral effect of APOBEC3A is exerted at viral DNA accumulation during *de novo* infection through a mechanism that may involve deamination. For the first time, our results indicate that APOBEC3A, rather than being devoid of inhibitory activity against HIV-1, plays an important role in blocking not only HIV-1, but more generally primate lentiviruses. This antiviral effect is specific to myeloid cells in which this factor is naturally expressed. Among the viral proteins capable of opposing APOBEC3A, we have found that Vpx, a protein coded by members of the SIV_SM_/HIV-2 lineage provides a partial protection against this factor by inducing its degradation. Overall, these results shed new light on the regulation of members of the APOBEC3 family in primary cells and open up new venues on the role that APOBEC3A, a restriction factor specifically expressed in myeloid cells, may play during viral pathogenesis.

## Introduction

The apolipoprotein B mRNA editing enzyme, catalytic polypeptide-like 3 family (APOBEC3s) comprises 6 members of highly related cytidine deaminases [1]. The prototype of the family, APOBEC3G (or A3G), has been identified on the basis of its ability to inhibit HIV-1 infection in the absence of the Vif protein [2]. In this case, the antiviral effect of A3G is exerted via its incorporation into virion particles in virus-producing cells. This incorporation leads to the deamination of newly synthesized viral DNA during the subsequent cycle of infection [3]–[6], although a non-deaminase dependent mechanism of inhibition has also been described [7], [8]. In the presence of Vif, A3G is targeted to an E3-ubiquiting ligase complex and is thus degraded in virus-producing cells [9]–[17]. Even if this seems the major mechanism with which the virus protects itself, retroviruses can use a Vif-independent manner that does not involve the degradation of A3G, but results in the exclusion of the protein from assembling viral particles [18]–[21]. In the case of HIV-1, Vif has also been proposed to promote structural changes in A3G that negatively affect its ability to be incorporated into virion particles [22], [23].

While this host-pathogen struggle takes place in producing cells, the role of the pool of A3G molecules present in target cells and thus welcoming incoming viral particles remains unclear and in large part unexplored [24], [25]. The possibility that APOBEC3 members might exert an inhibitory effect directly on incoming viruses is interesting, because in these steps Vif is absent (or present only in trace amounts in virion particles), so that the virus would be denuded of protection against them [26], [27]. A3G has been reported to block the early phases of HIV-1 infection of quiescent T cells and DCs, but this activity has been recently ascribed to more general modifications of the activation status of quiescent lymphocytes, rather than to a specific effect of A3G silencing during the early phases of infection [24], [25].

All the members of the APOBEC3 family coded by primates (A through H) exhibit antiviral properties, although their potency may vary (for review see [28], [29] and [30] for the specific case of A3H). The exception to this rule is APOBEC3A (A3A) that is to date the only member of the APOBEC3 family whose ectopic expression in established cell lines (HeLa or HEK293T cells) bears no consequence for retroviruses [21], [31]. APOBEC3A is a single-domain cytidine deaminase, it is capable of editing exogenous and endogenous DNA upon expression in HeLa cells and has been shown to be responsible for the strong editing activity observed in primary monocytes [32]–[35]. A3A is capable of inhibiting the replication of LTR and non-LTR retroelements [36]–[39], of Parvoviruses [36] and has more recently been shown to moderately inhibit Alpharetroviruses (more specifically the Rous Sarcoma Virus, RSV, upon incorporation into virion particles, [40]). Increased expression of A3A, along with other APOBEC3 members, has been observed in keratinocytes and skin of precancerous cervical biopsies of human papillomavirus (HPV)-positive patients, presence that has been correlated to an increased evidence of HPV editing [41]. Although certain studies suggest that the editing activity of A3A may play a role in the host cell genome integrity and advance the hypothesis that this activity may lead or contribute to cancerogenesis [34], the inhibition of retroelements and of the adeno-associated virus 2 (AAV2) seems independent from the deaminase activity of A3A [36]–[39].

In the case of HIV-1, no firm evidence links A3A to an antiviral activity in either producing or receiving cells, although one study advanced the hypothesis that the increased replication of HIV-1 in differentiated macrophages over monocytes could be due to their lower content in A3A [42]. Together with a few recent articles indicating that A3A could be preferentially expressed in cells of the myeloid lineage, these reports call for a deeper analysis of the relationship existing between HIV-1 and myeloid cells [43], [44].

Primary circulating blood monocytes, along with differentiated macrophages and dendritic cells (DCs) are key cells in immune responses acting both as sentinels of danger signals, as well as instructors for other cells of the immune system (reviewed recently in [45]–[47]. These cells represent an important target for HIV-1 replication and have been shown to serve both as a vehicle for its dissemination through the body, as well as a viral reservoir. However, myeloid cells are collectively more resistant to HIV-1 infection than other cell types, as for example activated lymphocytes. This resistance varies according to the differentiation status and stimulation present and appears at different steps of the viral life cycle. Among these steps, a major impairment occurs during the early phases of infection, as a number of laboratories including ours have established [48]–[55]. The exact nature of this resistance is unclear and likely to be multifactorial. However, a number of recent evidences point to the existence of cellular restriction factors that affect incoming virus specifically in the peculiar environment of myeloid cells [50], [56]. In this respect, two very recent studies identified one such factor in SAMHD1, whose alternative name is dendritic cells derived interferon γ-induced protein (DCIP) [57] and the deficiency of which causes the Aicardi-Goutières syndrome, a disease in which interferon responses are deregulated [58]. Despite the fact that SAMHD1 is expressed in different cell types, it seems to restrict lentiviral infection specifically in myeloid cells, probably highlighting the need for a particular cellular context, or for specific cellular partners [59], [60].

Given that previous reports indicated that A3A was preferentially expressed in CD14-positive monocytes [42]–[44], we tried to determine whether the pool of A3A molecules present in target cells could inhibit *de novo* infection of myeloid cells by HIV-1.

To this end, we first determined that A3A is the sole member of the APOBEC3 family specifically expressed in different myeloid cells. Then, using either ectopic expression of A3A in established HeLa cells or specific silencing in primary macrophages and DCs, we determined that A3A does indeed inhibit the early phases of infection specifically in myeloid cells. Indeed, silencing of A3A not only increases the susceptibility of target cells to HIV-1 during single round infectivity assays, but also augments the spread of R5 tropic HIV-1 viruses. This antiviral effect is mediated by the pool of A3A present in target cells and is exerted through a decrease in viral DNA accumulation. The identification of TC editing in vDNA produced during HIV-1 infection of myeloid cells suggests that this antiviral mechanism may at least in part be exerted via deamination of the viral genome. The inhibition mediated by A3A targets also SIV_MAC_ viruses, suggesting a conserved antiviral mechanism directed against primate lentiviruses. Finally, we provide evidence that Vpx, a protein coded by members of the SIV_SM_/HIV-2 lineage seems to provide partial protection against A3A by driving its degradation.

## Results

### A3A is the only member of the APOBEC3 family specifically expressed in primary blood cells of myeloid origins

**Figure 1:**
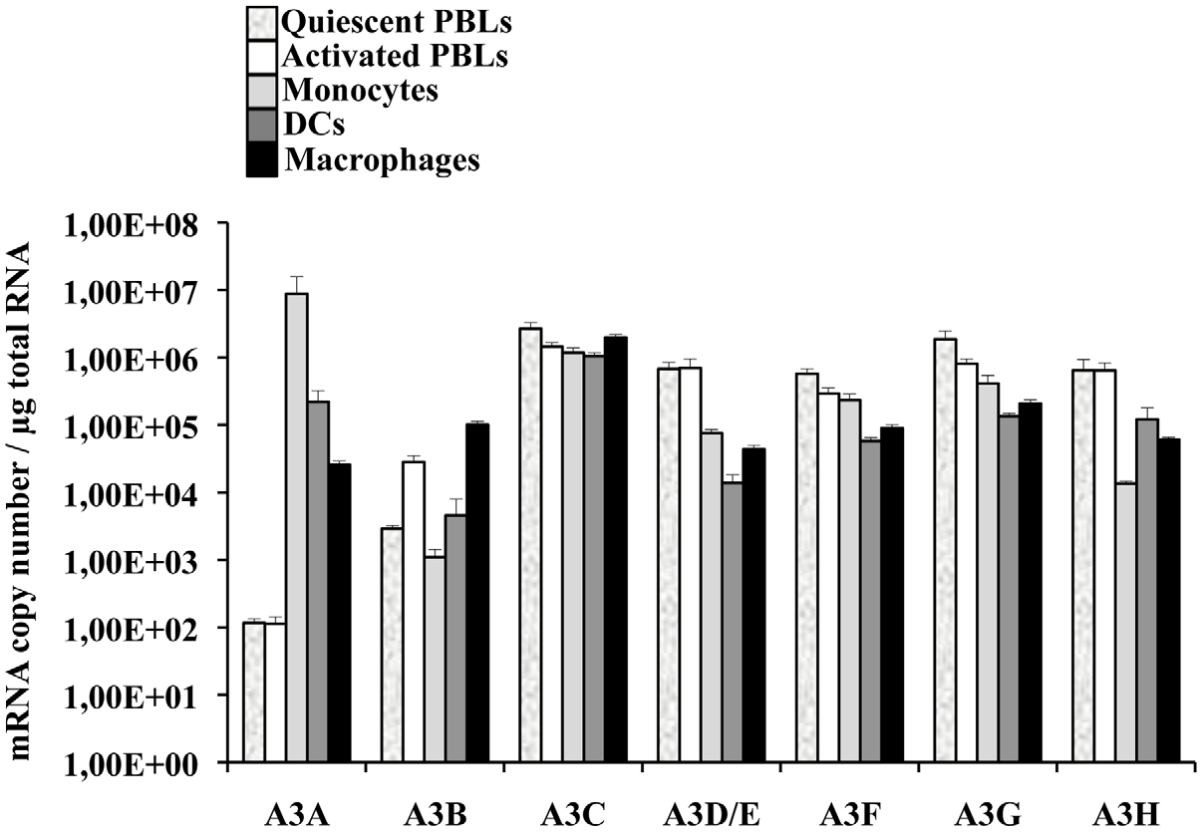
A3A is preferentially expressed in myeloid cells. Primary blood monocytes, monocyte-derived macrophages and DCs were compared to PBLs either quiescent or stimulated for 24 hrs with PHA/IL2 to determine the amount of mRNA of the different members of the APOBEC3 family using primers specific for each member, as already described in [44]. The graph presents results obtained from cells derived from 4 different donors.

A few reports have indicated that among circulating white blood cells, A3A was highly expressed in monocytes [42]–[44]. To determine more widely the pattern of expression of A3A and of the different members of the APOBEC3 family, quantitative RT-PCR was performed on quiescent or PHA-stimulated primary blood cells (PBLs, depleted of monocytes), monocytes, macrophages and dendritic cells (DCs) differentiated upon incubation of monocytes for 4 to 5 days with M-CSF and GM-CSF/IL4, respectively (Figure 1). Our analysis reveals that A3A is overexpressed by at least 100 fold in differentiated macrophages over PBLs, irrespectively of their activation status. This difference increases further in DCs and is the greatest in monocytes in which A3A is expressed over 5 logs more than in stimulated PBLs. The expression of the remaining APOBEC3 members did not display such cell type specific variations, with the exception of A3D/E that seems expressed at least 10 fold more in PBLs than in the myeloid cells tested. On the contrary, A3B and to a lower extent A3H are less expressed in non-stimulated monocytes than in stimulated PBLs, although their expression increased during differentiation into either macrophages or DCs. Overall, myeloid cells express consistent levels of all APOBEC3 members, but A3A is the only one whose expression is restricted to them. These results are in line with previous reports and extend them in the absolute quantification of the copy number of the different APOBEC3 members during the differentiation of circulating monocytes into macrophages and DCs. Of note, IFNα treatment increased the expression levels of A3A at both the mRNA and protein level, but the gradient of A3A expression did not change with respect to untreated cells: monocytes, DCs, macrophages and PBLs (from the highest to the lowest, Figure S1A and S1B). At the protein level, we confirm that A3A can be recognized after migration on an SDS-PAGE gel by the ApoC17 antibody initially raised against A3G [23] and Figure S2). In our hands, A3A can be detected as a doublet by WB. Detection of this doublet is however highly variable and unpredictable, probably influenced by the detection limit of A3A in a given experiment and by donor-to-donor variations. In any case, the antiviral effect of A3A seems not linked to the detection of an A3A doublet by WB.

In conclusion, these results indicate that if myeloid cells express comparable levels of APOBEC3 members, A3A is the sole member of the APOBEC3 family whose expression is restricted to cells of myeloid origins, among cells of hematopoietic origins.

### The expression levels of A3A specifically increase during HIV-1 spread in infected macrophages

**Figure 2:**
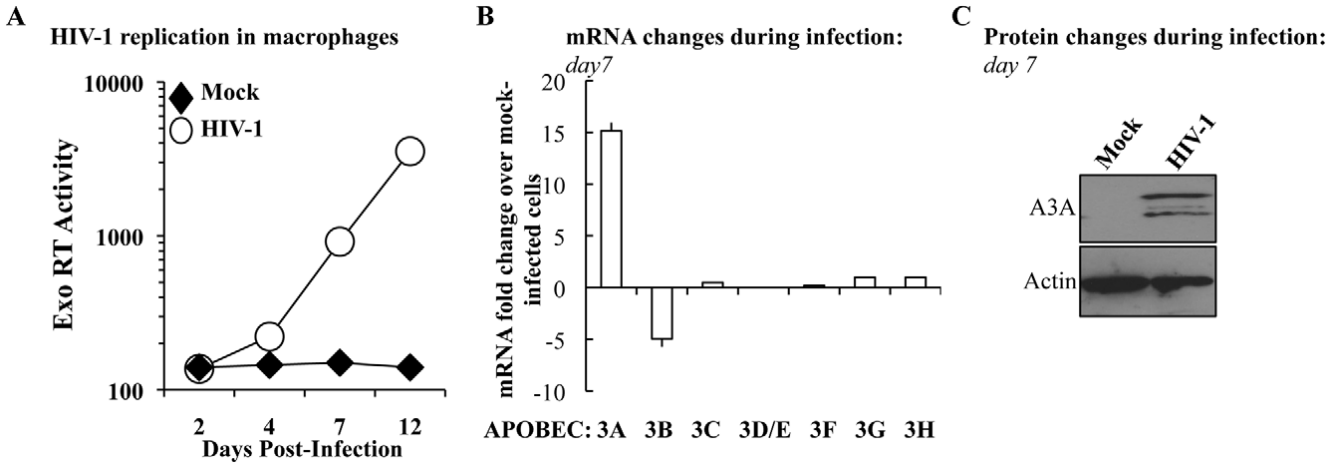
The expression of A3A is specifically increased during HIV-1 spread in infected macrophages. A) M-CSF-derived macrophages were infected with the R5 tropic HIV-1 strain Yu2 at a multiplicity of infection (MOI) of 0,05. Viral spread over time was measured by determining the amount of virion-associated RT activity released in the supernatant (exo-RT). Aliquots of macrophages were lysed at day 7 to determine variations in members of the APOBEC3 family at the mRNA (B) and, in the case of A3A, also at the protein level (C). The panels A and C present one representative experiment out of three, while the panel B presents the average values obtained with cells derived from 3 different donors.

To determine whether the levels of A3A were modulated during the course of HIV-1 infection, M-CSF-differentiated macrophages were infected with an R5 tropic HIV-1 virus (Yu2) and viral spread was assessed by determining the amount of virion-associated RT activity released in the cell supernatant (by exo-RT, Figure 2A). At day 7, when consistent viral spread had occurred, cell aliquots were lysed and the levels of APOBEC3 members analyzed at the mRNA level and, for A3A, also at the protein level by WB (Figures 2B and 2C, respectively). Spreading HIV-1 infection was associated to a consistent increase in A3A at both mRNA and protein levels. With the exception of A3B whose expression decreased during HIV-1 infection, the expression of the remaining members of the APOBEC3 family was not significantly modified. This increase was not due to the presence of type I interferon (IFNs) in the cultures, as assessed by the failure of supernatant derived from infected macrophages to inhibit the spread of a GFP-bearing Vesicular Stomatitis Virus in A549 cells (VSV, Figure S3). This assay is not only extremely sensitive, but allows the detection of the presence of all the subtypes of IFN α and β in the culture (in number of 13 and 2, respectively) [61], [62]. Although we cannot exclude the secretion of low levels of type I IFN and subsequent engagement of the IFN receptor, absence of IFN production during HIV-1 infection of dendritic cells has been recently reported [63]. Thus, although the expression of A3A can be induced by type I IFN [35], [43], [64], [65] and Figure S1), its expression during HIV-1 spreading infection of macrophages can also increase in a manner that is largely type I IFN-independent. To further support this argument, the expression of A3G, also modulated by type I IFN, is not significantly modified during infection. Thus, the upregulation of A3A observed here is likely to be part of a more complex response to viral infection that takes place in infected macrophages.

### A3A inhibits the early phases of HIV-1 infection specifically in myeloid cells

**Figure 3:**
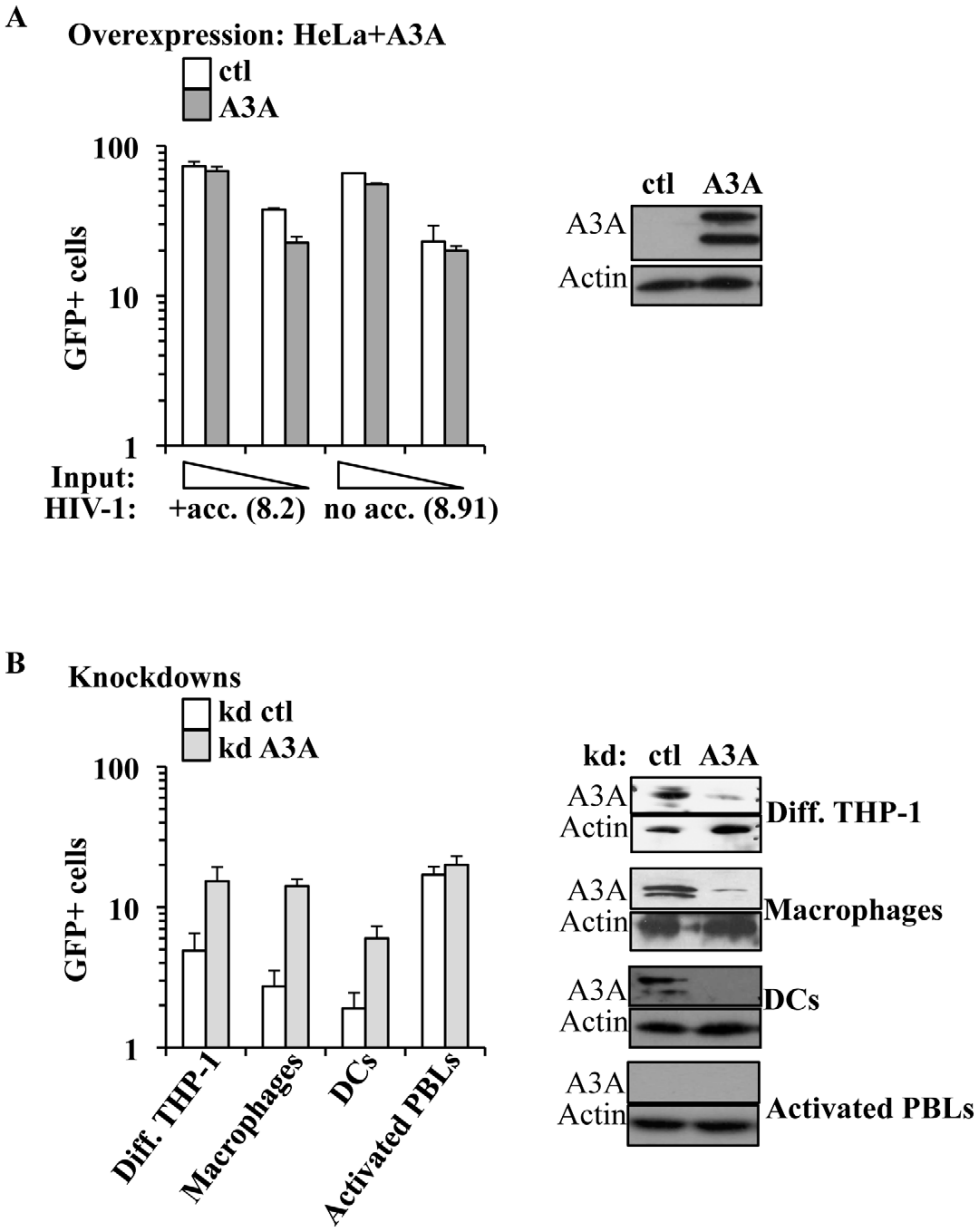
A3A inhibits the early phases of HIV-1 infection specifically in myeloid cells. A) HeLa cells were transfected with either control or HA-tagged A3A coding constructs and analyzed by WB or challenged 48 hours after transfection with different inputs of VSVg-pseudotyped complete or minimal HIV-1 vectors (i.e. coding or not viral non-structural proteins), carrying a GFP expression cassette in a single-round infectivity assay. The percentage of infected cells was determined 3 days later by flow cytometry. The graph presents averages from 4 different experiments. B) Control or A3A knockdowns were obtained upon LV-mediated transduction of PMA-differentiated THP-1 cells, macrophages, DCs and PHA/IL2-activated PBLs with an equal viral input of miR-shRNAs coding vectors. Four days later, stably silenced cells were analyzed by WB (right panel, presenting representative experiments) or challenged with VSVg-pseudotyped GFP-coding HIV-1 vectors (at MOIs comprised between 3 and 5). The graph presents data obtained from 3 to 6 independent experiments.

The main described anti-retroviral function of APOBEC3 members involves the pool of APOBEC3s molecules present in virus-producing cells. On the contrary, the role of the pool of APOBEC3 molecules present in target cells has been explored only for A3G and remains highly debated [24], [25]. Among the members of this family, A3A is to date the sole member devoid of known inhibitory activity against HIV-1 when overexpressed in established cell lines [21], [31]. In agreement with these previous results, single cycle infection of HeLa cells overexpressing an HA-tagged version of A3A (that is editing-competent, data not shown) did not significantly modulate the susceptibility of target cells to infection with both complete or minimal HIV-1 vectors (i.e. containing or lacking non-structural viral proteins, Figure 3A). Given that no differences were observed between complete and minimal vectors in the experiments described below, the formers were routinely used for their slight but consistent higher infectivity (data not shown and [66]).

Contrarily to what observed in HeLa cells, silencing of A3A increased the susceptibility of target myeloid cells to HIV-1 infection (Figure 3B). Silencing was established using either miR-shRNAs expressed upon HIV-1-vectors mediated gene transduction [67] or liposome-mediated transfection of siRNAs. Silenced cells were then challenged with GFP-coding HIV-1 vectors. Given that both silencing methods yielded similar results, the formers were routinely used, as they allowed for more potent silencing. Knockdown of A3A increased the susceptibility of all cells of myeloid origins tested to the challenge with HIV-1 by an average of 5 to 7 fold: THP-1 differentiated into macrophage-like cells upon incubation with PMA (Diff. THP-1), as well as macrophages and DCs (Figure 3B). As expected, knockdown of A3A exerted no effect on the infection of PHA-stimulated PBLs (in the presence or absence of IFNα, Figure 3B and Figure S4). This result was expected as A3A is barely expressed in both quiescent and activated PBLs even after IFNα treatment, if compared to myeloid cells. The absence of an effect of the A3A knockdown on the susceptibility of PBLs to HIV-1 infection suggests the lack of non-specific effects (i.e. the A3A target must be present for the observed phenotype). To further control the specificity of our knockdowns, quantitative RT-PCR analysis was carried out on mRNA preparations derived from miR-shRNA-silenced macrophages for all the members of the APOBEC3 family (Figure S5A). This analysis indicated a specific decrease in A3A mRNA, but not in the mRNAs of other members of the family. As further proof of the specificity of the A3A knockdowns, A3A was silenced in macrophages upon liposome-mediated transfection of siRNAs. These siRNAs where chosen to target sequences within the A3A mRNA that were distinct from those targeted by the miR-shRNAs. Again, knockdown of A3A yielded an increase in the infectivity of HIV-1 similar to the one observed for miR-shRNAs (Figure S5B).

Overall, these experiments reveal that A3A plays a specific antiviral role during the early phases of HIV-1 infection in cells of myeloid origins in which it is naturally highly expressed.

### A3A inhibits spread of HIV-1 in primary macrophages

**Figure 4:**
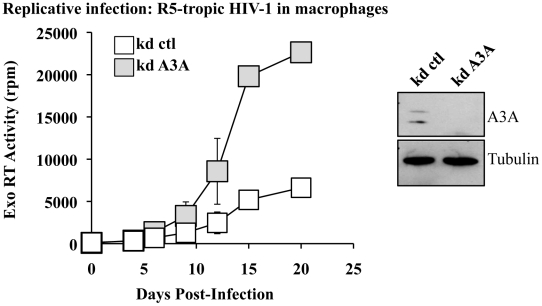
A3A silencing increases viral spread of an R5 tropic HIV-1 virus in macrophages. Macrophages stably silenced as above were infected with an R5 tropic HIV-1 virus (Yu2) at an MOI of 0,05. Viral spread was determined by exo-RT. The graph presents data obtained from 4 independent experiments, while the WB panels present the efficacy of A3A silencing at day 15 in one representative experiment.

To determine whether the antiviral effect of A3A could be observed also during replicative infection, silenced macrophages were challenged with R5 tropic HIV-1 (Yu2) at MOI of 0,05 and viral spread monitored by exo-RT activity (Figure 4). Even in this case, the specific silencing of A3A (shown in the WB side panel) increased viral spread in the culture. Although this result does not exclude an effect of A3A in virus-producing cells (the most classically described antiviral effect of APOBEC3s), this increase does correlate with the one observed during the early phases of infection in single round infectivity assays, indicating that A3A inhibits viral spread in macrophages. In light of the data presented in Figure 2B indicating an increase of A3A during spreading infection of HIV-1, we can hypothesize that the effect of silencing of A3A is mitigated by the overexpression of A3A observed during spreading HIV-1 infection. Similar experiments were attempted in DCs, but were abandoned due to the high mortality of silenced DCs upon viral challenge. This higher mortality is independent from the identity of the silenced gene, as it is observed in control and A3A knockdowns alike and does not seem specific for HIV-1.

### A3A impairs the accumulation of vDNA during the early phases of infection, but does not affect the kinetics of accumulation of infectious vDNA

**Figure 5:**
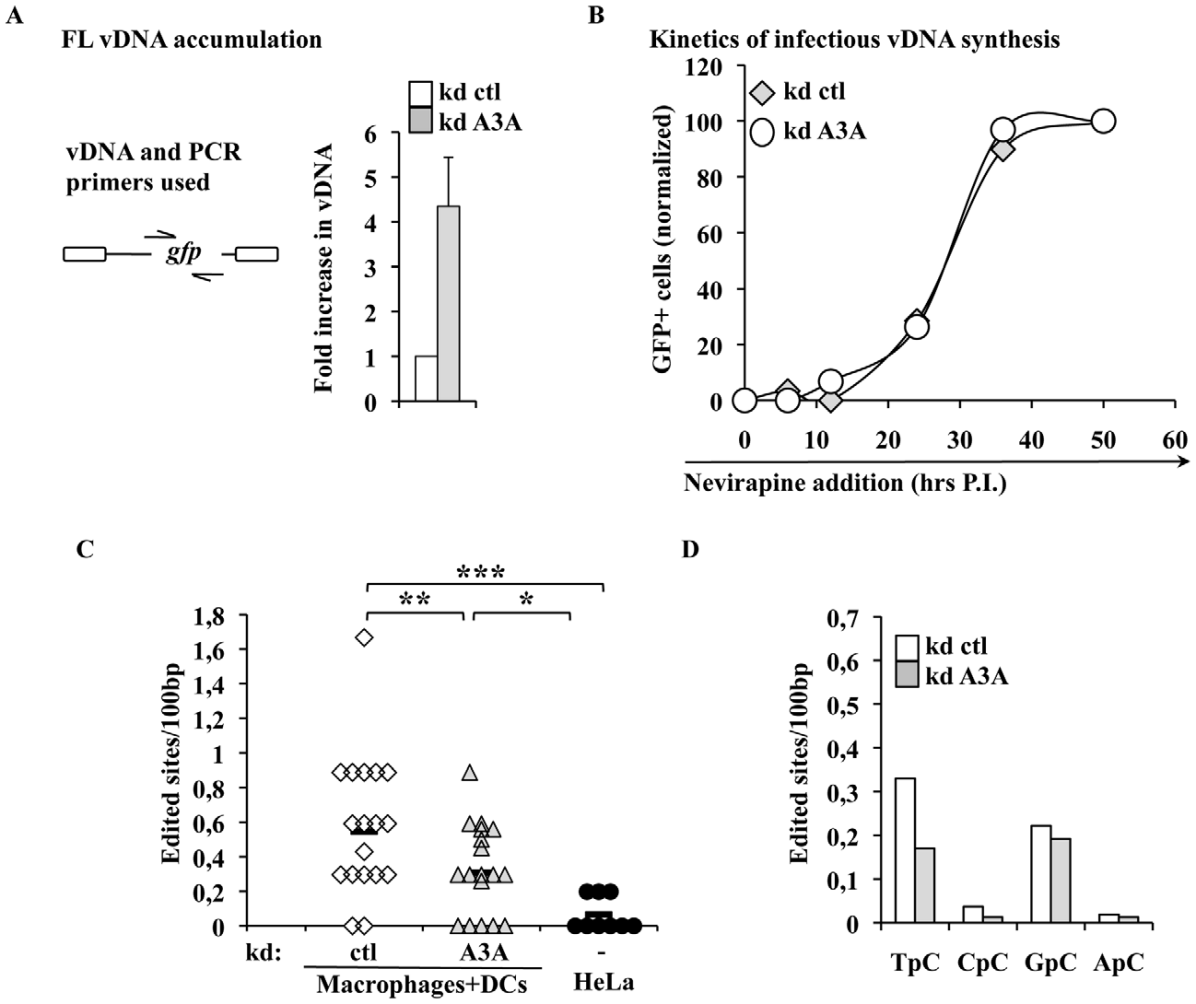
A3A impacts the accumulation of vDNA, but not the kinetics of reverse transcription of infectious vDNA and modulates the levels of TC editing of vDNA produced during the infection of myeloid cells. A) Control and A3A-silenced macrophages were challenged with an equal viral input of GFP-coding HIV-1 virus and cells were lysed at 24 hours post-infection for qPCR analysis with primers specific for the GFP sequence present uniquely on the challenge virus. The scheme presents the relative position of PCR primers on the HIV-1 genome. Control infections were carried out in the presence of RT-inhibitors. Given values are normalized for actin and subtracted for those obtained upon infections carried out in the presence of RT inhibitors (Nevirapine and AZT at 20 µg/ml). B) The kinetics of infectious vDNA accumulation in macrophages silenced or not for A3A were determined as in [48]. Briefly, the accumulation of infectious genomes, defined as those resulting in a GFP-positive cell, is determined upon addition of the RT inhibitor Nevirapine at different times post infection. The percentage of vDNA molecules completed prior to the addition of the inhibitor at each time point is determined 3 days post infection upon normalization to control infections carried out in the absence of RT inhibitor. This assay yields a measure of the speed at which infectious vDNA accumulates, as described in [48]. The graph presents a representative experiment out of 3. C) vDNA produced upon infection of HeLa cells or of primary silenced macrophages was extracted 24 hours post infection, amplified and individual clones, represented by symbols, sequenced. The number of cytidine edited in each sequence with respect to the reference is shown here. Sequences were obtained from 4 independent experiments. Lines represent averages in each sample. Asterisks indicate statistically significant differences after a Student t test (* p<0,05;** p<0,01;*** p<0,001). D) Analysis of the dinucleotide motif of edited cytidine of the sites shown in C.

To identify the step affected by the depletion of A3A in target cells, silenced macrophages were lysed 24 hours post infection and the accumulation of vDNA analyzed by qPCR. The use of an HIV-1 based miR-shRNA vector precluded the analysis of all the viral DNA products synthesized over the course of infection. However, the presence of GFP in the challenge HIV-1 vector enabled the analysis of vDNA products obtained after the minus strand strong stop, which represent one of the major restrictive step during the infection of myeloid cells. Under these conditions, silencing of A3A increased the accumulation of vDNA by 4 to 5 fold (Figure 5A), in agreement with the increase observed in infectivity. While the analysis of vDNA products by PCR yields quantitative data on their accumulation as a whole, this technique does not reveal the kinetic behavior of truly infectious vDNA. Infectious vDNA constitutes a minor fraction of the total vDNA produced during infection [68], a phenomenon that is exacerbated in primary cells more resistant to infection. Thus, determining the behavior of these genomes represents an important additional parameter during the early phases of infection. To determine the kinetic behavior of infectious vDNA and more specifically to determine how fast it was completed during infection, we employed a technique we had previously described [48]. In this setup, infectious viral genomes are defined as those capable of expressing the viral-coded GFP reporter and their accumulation over time is determined by arresting the reverse transcription process at different times post infection through the addition of the RT inhibitor Nevirapine. The percentage of GFP-positive cells at each time point is then determined by flow cytometry 3 days post infection and values are graphed after normalization to control infections carried out in the absence of RT inhibitors. This assay yields a kinetic measurement of the speed at which completion of infectious vDNA is carried out. In this respect, the assay does not yield information on the total amount of vDNA synthesized during infection as in Fig. 5A, but describes another parameter which is the speed at which infectious vDNA is produced. When control and A3A-silenced macrophages were thus analyzed, no significant changes were observed in the kinetics of reverse transcription of HIV-1 infectious genomes (Figure 5B). These results indicate that A3A does not modify the kinetics of reverse transcription, but affects the overall amount of vDNA synthesized. This decrease is of the same amplitude of the effect observed in viral infectivity.

### The intracellular levels of A3A modulate the levels of TC editing found in vDNA produced during infection of myeloid cells

Inhibition of AAV or retroelements by A3A seems to follow a deaminase-independent mechanism. To determine whether this was the case here, vDNA products accumulated during infection of macrophages were amplified, cloned and sequenced in search of potential cytidine editing. HIV-1 vDNA produced in macrophages exhibited a significantly higher proportion of edited cytidine with respect to vDNA synthesized in HeLa cells (Figure 5C). This deamination signature depended at least in part from A3A, since the number of edited sites decreased upon silencing of A3A. When the dinucleotide motifs surrounding the edited cytidine were examined, a minority was found to be CC (described as specific of A3G), while more than a half were TC (described to be preferentially recognized by the remaining A3 members) (Figure 5D). Cytidine deamination of the latter TC motif depended from A3A as their proportion specifically decreased upon A3A silencing. Overall, these results indicate that a small yet detectable cytidine editing signature is present in vDNA produced during the infection of myeloid cells, a portion of which is clearly influenced by the intracellular levels of A3A.

#### The antiviral effect of the A3A pool present in target cells is conserved among primate lentiviruses

**Figure 6:**
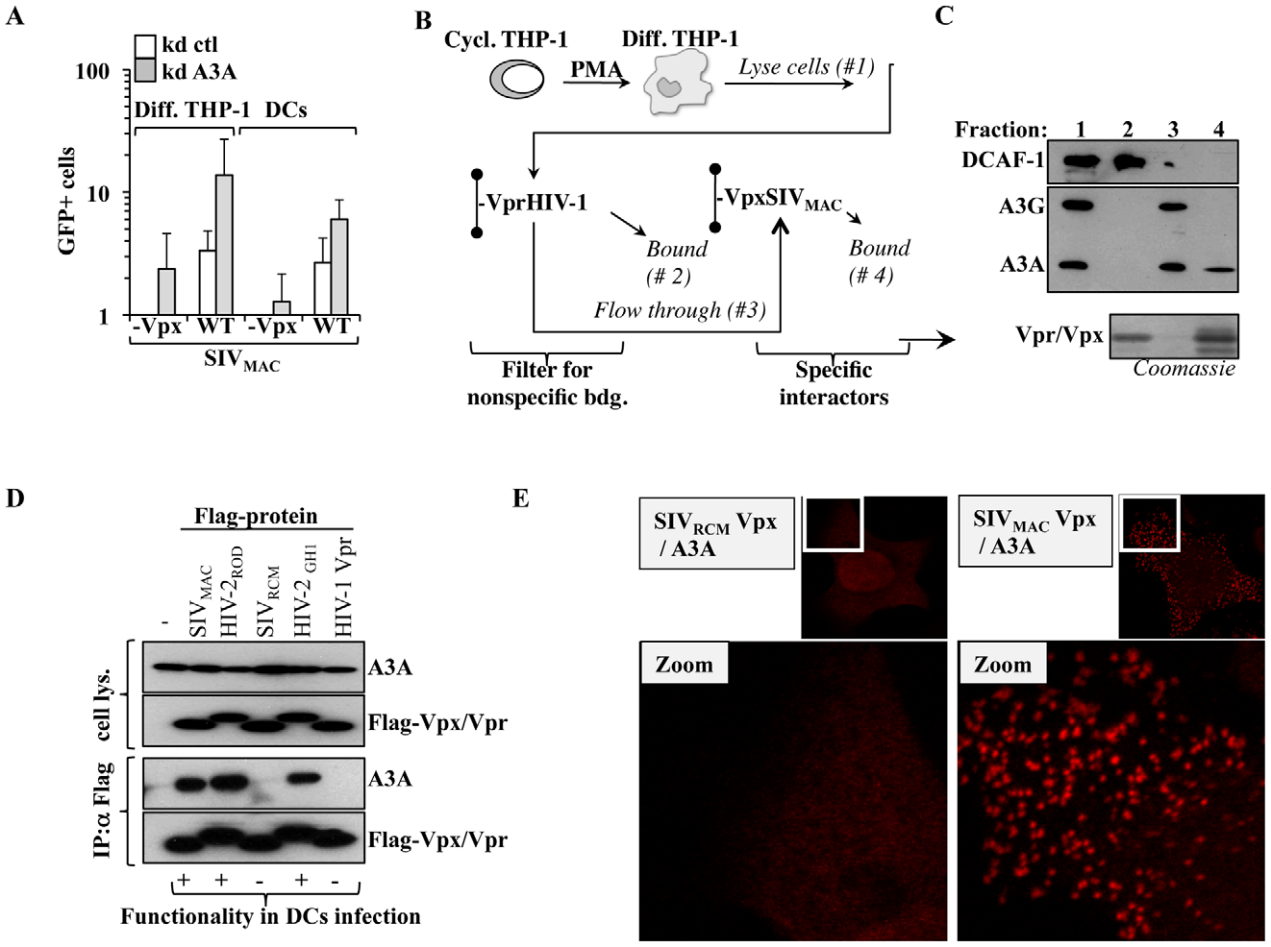
A3A affects more generally primate lentiviruses. SIV_MAC_ Vpx provides partial protection against A3A and functionally interacts with it. A) Control and A3A knockdown cells were challenged with an equal amount of SIV_MAC_ vectors carrying or not Vpx and the results of the infection were analyzed 3–4 days after by flow cytometry. B) Scheme used to detect the interaction between Vpx and endogenous A3A expressed in PMA-treated THP-1 cells. Briefly, nichel columns containing purified His-tagged HIV-1 Vpr (non functional during the infection of primary myeloid cells) was used to deplete the cell lysate of non specific interactors prior to its addition to an identical column containing SIV_MAC_ Vpx (functional in the infection of these cells). C) Retained proteins were analyzed by WB with the indicated antibodies, or analyzed by Coomassie staining for the Vpx/Vpr inputs. D) HEK293T cells were transfected with the indicated plasmids coding for Vpx and Vpr proteins whose ability to rescue the infectivity defect of Vpx-deficient SIV_MAC_ vectors we had previously determined [50]). Co-immunoprecipitations were performed using anti-Flag antibody-coupled beads. E) HeLa cells were similarly transfected then fixed and stained for DuoLink immunofluorescence experiments. A positive signal in DuoLink experiments requires the spatial proximity between two proteins (less than 40 nm) for a signal to appear in the form of dots. Representative experiments are shown here.

To determine whether A3A is a specific inhibitor of HIV-1, or if it exerts a conserved antiviral function directed against primate lentiviruses, A3A or control-silenced differentiated THP-1 cells and DCs were challenged with SIV_MAC_ vectors. Given that we have previously determined that the non-structural protein Vpx plays an important role during the infection of myeloid cells, Vpx-positive and deficient SIV_MAC_ viruses were compared side by side (Figure 6A). Silencing of A3A resulted in an increase in the infectivity of WT SIV_MAC_ viruses in both differentiated THP-1 cells and DCs, indicating that A3A targets not only HIV-1, but more generally primate lentiviruses. A positive effect of A3A silencing was also observed in the infectivity of SIV_MAC_ vectors devoid of Vpx, that display a strong defect during the infection of myeloid cells. In differentiated THP-1, the infectivity of SIV_MAC_ Vpx-deficient viruses approached –albeit not equaled- that of the WT in control silenced cells. In DCs, in which SIV_MAC_ Vpx-deficient viruses display a drastic infectivity defect, silencing of A3A allowed the detection of infection clearly above the background, although not to the same levels of WT SIV_MAC_. These results suggest that the depletion of A3A may contribute to relieve the defect of Vpx-minus viruses. This result is of interest for two reasons. First because we and others have previously determined that Vpx is required for the completion of the early phases of infection specifically in myeloid cells in a manner that may involve the degradation of an unknown cellular restriction factor [50], [56], [67], [69]–[71]. Second because A3A has been previously shown to interact with SIV_MAC_ Vpx upon overexpression in HeLa cells [72]. Thus, we decided to pursue the characterization of the relationship between Vpx and A3A further.

#### SIV_MAC_ Vpx associates functionally to A3A

His-tagged SIV_MAC_ Vpx and HIV-1 Vpr were purified on nichel columns and used to pull down endogenous A3A expressed in PMA-treated THP-1 cells. We have already determined that although these proteins are structurally related, only SIV_MAC_ Vpx exerts a strong positive effect during the early phases of infection of myeloid cells [50]. HIV-1 Vpr was used to filter the lysate obtained from differentiated THP-1 of non specific interactors, prior to binding to a second column containing SIV_MAC_ Vpx (Figure 6B). The different fractions were then subjected to WB analysis for the indicated proteins (Figure 6C). The DDB1 and CUL4-associated factor 1 (DCAF1) adaptor was examined, as it has been shown to associate to both HIV-1 Vpr and Vpx proteins derived from almost all SIV and HIV-2 strains tested [56], [67], [69], [73]–[78], although the relevance of this binding for the functions of Vpx remains controversial [67], [69], [70]. As expected, DCAF1 was almost completely retained by the HIV-1 Vpr column, thus validating our filtration scheme. On the contrary, A3G and A3A, recognized by the same antibody and distinguishable by size upon migration in an SDS-PAGE gel, were not. Upon binding of the flow-through to the SIV_MAC_ Vpx column, only A3A was retained indicating that it bound endogenous A3A but not A3G in differentiated THP-1 cells. To characterize this interaction further, HEK293T cells were transfected with several Flag-tagged Vpr and Vpx proteins of known functionality during the early phases of lentiviral infection in primary myeloid cells along with a construct coding HA-Tagged A3A (Figure 6D). Cells were lysed and co-immunoprecipitations were carried out using anti-Flag antibody-coated beads. Under these conditions, A3A co-immunoprecipitated only with Vpx proteins derived from SIV_MAC_, HIV-2_ROD_ and HIV-2_GH1_, but not with SIV_RCM_ Vpx nor with HIV-1 Vpr. Thus, A3A associates with proteins that exert a positive function during the early phases of infection of myeloid cells, but does not associate to non-functional ones.

To provide further evidence of the A3A-Vpx interaction with an independent technique, DuoLink experiments were carried out. In DuoLink-based immunofluorescence, the spatial proximity between two proteins (≤40 nm) is required for the generation of a fluorescent signal that is amplified *in situ* and that appears in the form of a dot upon image analysis. Very simplistically, instead than being directly coupled to a fluorophore, secondary antibodies are coupled to 2 distinct single-stranded oligonucleotides that, when in close proximity, interact with a substrate oligonucleotide that serves as the basis for fluorescence development. Thus, the DuoLink technique does not show the intracellular distribution of two proteins, but reveals if and where they might interact in the cell (Figure 6E). Under these conditions, a diffused background signal was obtained in HeLa cells stained solely with 2ndary antibodies or upon transfection of A3A with the non-functional SIV_RCM_ Vpx (in our experience, this diffuse staining is the normal background obtained with DuoLink). On the contrary, a clear positive signal in the form of dots was obtained when A3A was co-transfected with SIV_MAC_ Vpx. These dots were mostly localized in the cytoplasm of transfected cells, supporting the notion that A3A and Vpx interact in this intracellular location and corroborating our previous findings indicating that Vpx is significantly -although not exclusively- localized in the cytoplasm of expressing cells. Of note, the distribution of Vpx and A3A did not change when these proteins were expressed alone or together, as determined by standard confocal microscopy, indicating that this interaction does not induce a massive relocalization of either proteins (not shown).

All together, these results indicate that A3A associates functionally with Vpx.

### SIV_MAC_ Vpx specifically degrades A3A in HeLa cells and in primary DCs

**Figure 7:**
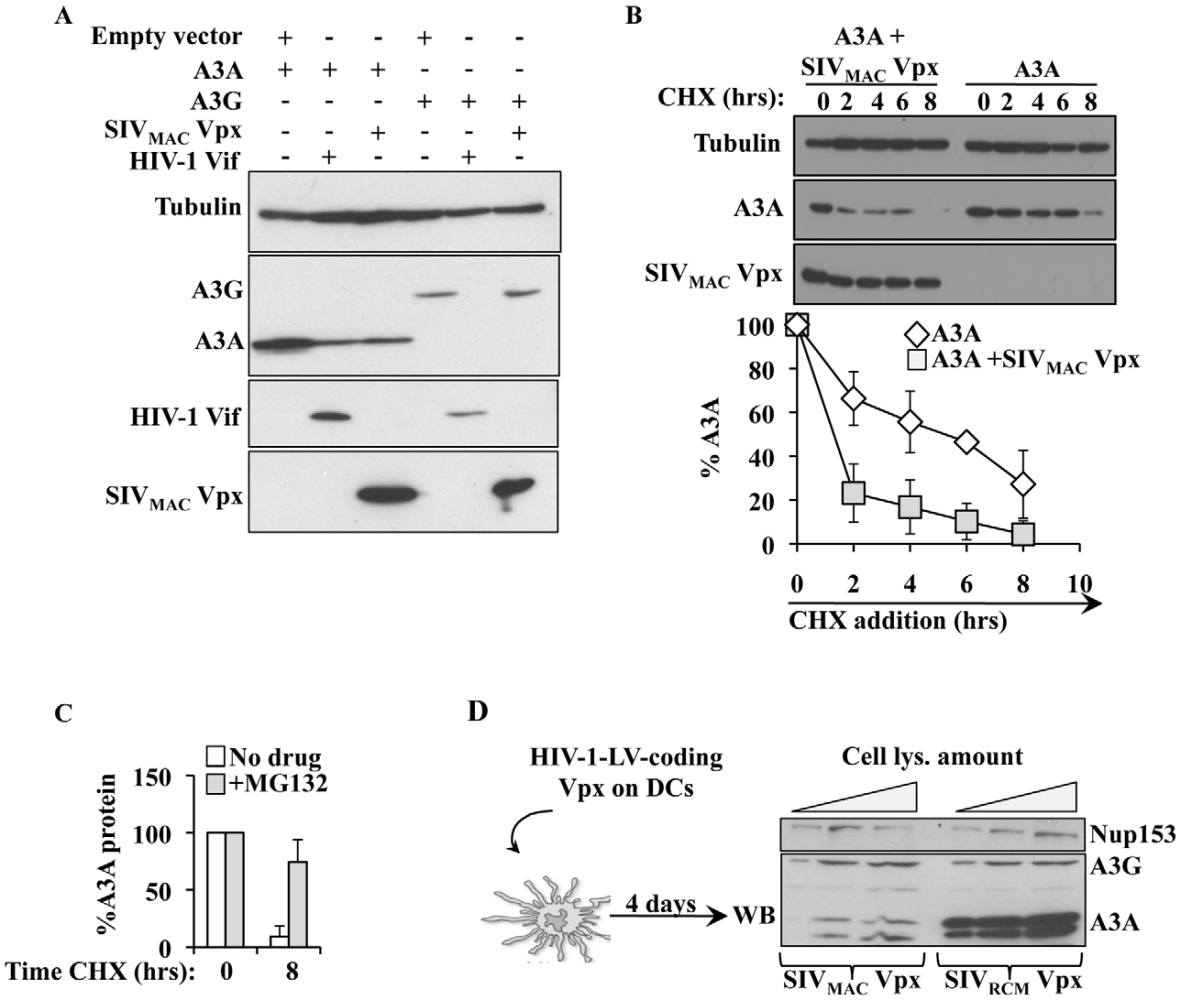
SIV_MAC_ Vpx degrades A3A but not A3G in both HeLa cells and DCs. A) HEK293T cells were transfected with the indicated plasmids then lysed for WB analysis 48 hours post transfection. B) As in A, but Cyclohexamide (CHX) was added onto cells at the indicated time prior to cell lysis and WB analysis. C) As in B, with cells incubated in the presence or absence of the proteasome inhibitor MG132 (10 µg/ml) for 8 hours prior to cell lysis. D) Scheme and results used here to determine the degradation of A3A in DCs. HIV-1 vectors coding Vpx proteins derived from SIV_RCM_ and SIV_MAC_ (non-functional and functional during myeloid cells infection, respectively) were produced by transfection of HEK293T cells, normalized by protein content and used to transduce DCs. Vpx-expressing DCs were lysed 4 days post infection and analyzed by WB.

A previous report indicated that SIV_MAC_ Vpx could degrade A3A [72]. To determine whether this was the case, A3A and A3G were co-transfected along with HIV-1 Vif and SIV_MAC_ Vpx in HeLa cells (Figure 7A). As expected, HIV-1 Vif decreased the steady state levels of A3G and A3A, although this effect was less pronounced for A3A. On the contrary, SIV_MAC_ Vpx affected only A3A, but not A3G. When the stability of A3A was determined, a clear decrease in its stability was observed upon co-expression of SIV_MAC_ Vpx (Figure 7B). This degradation seems to involve a proteasome dependent mechanism since degradation of A3A by Vpx is significantly blocked upon incubation with the proteasome inhibitor MG132 (Figure 7C).

To determine whether Vpx was also able to degrade endogenous A3A in myeloid cells, SIV_RCM_ and SIV_MAC_ Vpx proteins were expressed in DCs after lentiviral-mediated gene transduction. Briefly, HIV-1 vectors coding the 2 Vpx proteins were produced in 293T cells, normalized by protein content and used to transduce DCs. Under these conditions, Vpx was not incorporated into HIV-1 particles and thus did not affect the infectivity of these vectors in DCs (data not shown). When DCs were analyzed 4 days after transduction, a clear decrease in the levels of A3A was observed in DCs expressing SIV_MAC_ Vpx, but not in DCs expressing SIV_RCM_ Vpx. This decrease was specific for A3A, as the levels of A3G were unaffected by the expression of Vpx proteins.

Overall, these results indicate that SIV_MAC_ Vpx may partially protect SIV_MAC_ viruses from the negative effects of A3A by inducing its degradation during the early phases of infection.

## Discussion

In the present study we reveal a novel role for A3A in controlling HIV-1 replication specifically in myeloid cells. Cells belonging to this lineage express all members of the APOBEC3 family, but A3A is the only one restricted to myeloid cells among the white blood cells targeted by HIV-1 *in vivo* and is also the sole member whose expression augments during spreading infection of primary macrophages. The results presented here indicate that the specific silencing of A3A in differentiated THP-1 cells (that mimic macrophages upon differentiation), primary macrophages and DCs increases their susceptibility to *de novo* HIV-1 infection, while on the contrary silencing in primary PBLs or ectopic expression of A3A in established cell lines exerts no effect on these phases. Thus, these results indicate that the antiviral effect of A3A is common to cells of myeloid origins. We have been so far unable to silence A3A in monocytes in a robust manner that also preserves their non-stimulated phenotype, so that the effect of A3A on HIV-1 could not be explored. However, in light of its extremely high expression, we may surmise that A3A hampers HIV-1 in non-stimulated monocytes, as suggested previously [42].

Overall, our findings are in agreement with past studies indicating that A3A does not exert an antiviral activity in HeLa or HEK293T cells [21], [31], and extend them by revealing that this property is explicit only in cells in which A3A is naturally expressed, i.e. myeloid cells.

While the restricted expression of A3A indicates a tight transcriptional regulation, the lack of antiviral activity upon ectopic expression in HeLa cells suggests that the mere expression of A3A is not sufficient to confer an antiviral state to recipient cells. This observation suggests that A3A may be regulated also at the post-translational level through modifications that affect its activity, localization, association to co-factors, processivity or all these things together. This cell type dependent regulation is not unprecedented among members of the APOBEC3 family, as an as yet unidentified inhibitory activity has been recently proposed to regulate the editing activity of A3G in T cells [79]. In the case of A3A, these different levels of control may be extremely important to regulate its activity and cytotoxicity. In our hands, the stable expression of A3A is particularly cytotoxic (data not shown), an effect that is likely linked to its ability to edit the cellular genome and to induce a DNA damage response that is followed by cell cycle arrest in A3A-overexpressing cell lines [32], [34], [35]. However, myeloid cells express constitutively high levels of A3A and must have devised manners to protect themselves from these negative effects. Since all the myeloid cells tested here are non-dividing, it is possible that this state makes them less susceptible to editing of the cellular genome or to a DNA damage response than cycling cells. Alternatively, and not mutually exclusive with the hypotheses mentioned above, a specific post-translational regulation might protect myeloid cells. Such modifications, and/or the association with other cellular components may strongly influence the ability of A3A to recognize its target for example by shaping the single-stranded DNA docking groove of A3A, as identified in [80].

The results presented in this study indicate that the pool of A3A present in target cells is directly capable of inhibiting incoming viruses. This possibility had been raised before in quiescent T cells and DCs for A3G, although a recent report tempered enthusiasm over the function of APOBEC3 members during these phases [25]. Our results demonstrate that the specific knockdown of A3A positively impacts the early phases of HIV-1 infection, as clearly observed using vectors capable of a single round of infection. An antiviral effect at this step is conceptually interesting because Vif, that normally inhibits APOBEC3s during the phases of virus production, is absent in virion particles [26], [27]. As a consequence, incoming virus may be largely deprived of one of its major protection against APOBEC3 members present in recipient cells. Indeed, as a further proof that Vif cannot influence the early phases of infection, use of minimal HIV-1 vectors deficient in Vif, did not modify the results obtained here (data not shown). At present, we ignore whether this inhibition during the early phases of infection is specific to the combination A3A-myeloid cells, or if it is more generally present in other primary cells. Despite the fact that a number of studies have characterized the lack of effect of APOBEC3 members during the early phases of infection, rare are those that examined this question in the natural targets of HIV-1 replication. In light of the results presented here, we believe this issue deserves further investigation.

A3A inhibits HIV-1 during reverse transcription, the step that a number of laboratories including ours have determined as a major restriction during the infection of primary myeloid cells [48]–[55]. The process of reverse transcription is extremely sensitive to the intracellular environment and multiple factors have been shown to affect it (core-stability, viral protein processing, dNTPs levels, possibly intracellular trafficking and so forth). So, in principle, A3A could inhibit this process by affecting any of these parameters either more potently than other APOBEC3 members or else more specifically in myeloid cells in which these steps take place at a slower pace with respect to other cell types. Viral DNA produced upon infection of primary myeloid cells presents cytidine editing in higher levels than the one synthesized in HeLa cells. More than 50% of edited cytidines are present within a TC dinucleotide recognized by all members of the APOBEC3 family with the exception of A3G, while most of the remaining edited sites are within a GC dinucleotide that does not seem to be specific for APOBEC3s. Only a minority of edited cytidine is present in a CC dinucleotide context specific to A3G (10 fold less than TC). Among the different sites, the levels of TC editing are specifically influenced by the intracellular levels of A3A, indicating it as a major responsible for TC editing in myeloid cells. The amount of cytidine editing observed here is small and unlikely to account for the antiviral effect of A3A observed here. However, this low amount of editing may represent only the detectable fraction of the total edited vDNA, as edited vDNA may be efficiently removed or repaired in myeloid cells. Given that detection of this small amount of vDNA editing is challenging, previous reports indicating its absence upon A3A inhibition of LINE-1 retrotransposons and AAV2 may mean that either editing is in these cases lower than what observed here, or else that A3A can exert its antiviral effect independently from deamination [36]–[39].

Although our results reveal that A3A plays a role directly in target cells, the finding that A3A is a cell type specific restriction factor suggests that A3A may also exert the most classical of the antiviral activities of APOBEC3s, namely the one of being incorporated into HIV-1 virions and then deaminate newly synthesized vDNA. In light of the exquisite cell type dependent regulation of the activity of A3A this possibility is highly likely and in this case HIV-1 Vif that does degrade A3A, although less efficiently than A3G, may protect the virus against A3A as it does against other APOBEC3s.

The features of HIV-1 inhibition by A3A seem essentially conserved among primate lentiviruses, as the depletion of A3A increases also the infectivity of SIV_MAC_. However, we have noticed that Vpx-deficient SIV_MAC_ viruses are more responsive to A3A depletion than WT ones, a phenotype that is more marked in differentiated THP-1 cells, although less so in DCs. This may relate to the more drastic infectivity defect of Vpx-deficient SIV_MAC_ viruses in the latters. Vpx is to date the sole non-structural viral protein coded by primate lentiviruses capable of affecting the early phases of infection specifically in myeloid cells [50]. In the past, we have hypothesized that this could be due to the block of a restriction factor specifically expressed in these cells [50], [56]. In our hands, Vpx proteins interact functionally with A3A using three different methods: binding of endogenous A3A obtained from the lysate of differentiated THP-1 cells, co-IP after co-expression and DuoLink immunolocalisation that requires the physical proximity of two proteins for a positive signal to develop (less than 40 nm). This interaction leads to the degradation of A3A, but not of A3G, in HeLa cells and more importantly in primary DCs. Thus, our results are in agreement and extend a past study indicating that Vpx functionally interacts and degrades A3A after co-expression in established HeLa cells [72]. In the past, we have shown that SIV_MAC_ Vpx modifies the susceptibility of myeloid cells to lentiviral infection with both cognate and non-cognate viruses (as HIV-1) [50], [81]. Under these conditions, the major effect of Vpx on the early phases of HIV-1 infection was exerted on the speed at which viral DNA accumulated, a phenomenon that we had hypothesized to protect vDNA from the negative effects of a particularly hostile intracellular milieu [50]. Given that knockdown of A3A does not affect the kinetics of vDNA formation following HIV-1 infection, we believe that A3A may not be the sole effector counteracted by Vpx (Figure S6). However, the fact that A3A silencing increases in a more dramatic manner the infectivity of Vpx-deficient viruses, as well as the fact that A3A is degraded upon expression of Vpx in HeLa cells and DCs indicates that A3A is an important target of Vpx during the infection of myeloid cells. Two recent studies identified SAMHD1 as a cellular factor that is specifically bound and degraded by Vpx [59] [60]. Contrarily to A3A, SAMHD1 is expressed in cells other than myeloid cells, as its isolation from HEK 293T cells indicates [59]. However, its antiviral effect is apparent mainly in myeloid cells, arguing for the need of a particular environment or for specific cellular co-factors. In light of the results presented here, A3A whose expression is truly restricted to the myeloid lineage (among cells of hematopoietic origin) may be a co-factor in the antiviral action of SAMHD1 or conversely, SAMHD1 may be an important cofactor in the action of A3A. The fact that Vpx seems to degrade two factors that hamper the early phases of infection specifically in myeloid cells evokes the possibility that they may act on the same pathway.

In conclusion, our results reveal a novel role for A3A in inhibiting the *de novo* infection of myeloid cells with HIV-1 and more generally with primate lentiviruses. In light of the extreme importance of myeloid cells during HIV-1 pathogenesis, these results call for a more ample evaluation of the impact of A3A during HIV-1 pathogenesis in cells that play a key instructive role in immune system responses.

## Materials and Methods

### Cell culture, cytokines and antibodies

293T and HeLa cells were maintained in Dulbecco's Eagle Modified Medium (DMEM) supplemented with 10% of fetal calf serum (FCS) and 100 U of penicillin/streptomycin. The THP-1 monocytic cell line was maintained in RPMI1640 medium supplemented with 10% FCS, HEPES 10 mM, 0,05 mM β-mercaptoethanol and 100 U of penicillin/streptomycin. THP-1 cells were differentiated for 24 hours in presence of 100 ng/ml of phorbol-12-myristate-13-acetate (PMA, SIGMA) to induce their differentiation into macrophage-like cells. Primary blood human monocytes and peripheral blood lymphocytes (PBLs) were purified from the blood of healthy donors obtained at the blood bank of Lyon (EFS-Lyon), as described before [67], yielding cell populations of purity greater than 95%. Monocytes were differentiated into macrophages or immature dendritic cells (DCs) upon incubation for 4 to 5 days in RPMI1640 complete medium supplemented with 100 ng/ml of MCSF or 100 ng/ml of GM-CSF/IL-4, respectively (AbCys). PBLs were either left in the absence of stimulus (quiescent) or stimulated with phytohemmagglutinin (PHA, SIGMA at 1 µg/ml) and IL2 (150 U/ml, AIDS Reagents and Reference Program of the NIH) for 24 hrs prior to infection. When indicated cyclohexamide (CHX, SIGMA) was added at a concentration of 100 µg/ml to arrest cell translation and MG132 (SIGMA) was added for 8 hrs at 10 µg/ml to block proteasomal degradation. When indicated IFNα2A (Tebu-Bio) was used at a final concentration of 1000 U for 24 hrs prior to infection. Anti-Flag, anti-HA, anti-actin and anti-tubulin monoclonal antibodies were purchased from SIGMA while, anti-HIV-1 Vif and anti-A3G Apo-C17 antibodies that recognize A3G and also A3A (recognized for its smaller size) were obtained from the AIDS Reagents and Reference Program of the NIH.

To determine the amount of type I interferons released in the supernatants of infected macrophages, we adopted a well established method that allows the simultaneous detection of multiple α and β IFN subtypes thanks to their ability to functionally inhibit the infection of target A549 cells with a GFP-bearing VSV. VSV is replicative virus that is extremely susceptible to the presence of type I interferons. Briefly, supernatant dilutions harvested from macrophage cultures were incubated with A549 cells prior to challenge with GFP-bearing VSV. The percentage of GFP-infected cells is assessed 24 hrs later by flow cytometry and the amount of type I IFN (U/ml) obtained against a standard curve. Plasmacytoid DCs (pDCs obtained by positive selection, Miltenyi) purified from the blood of healthy donors were used as control.

### DNA constructs and viral production

The plasmids coding HA-tagged A3A and A3G were a kind gift of Dr. Kenzo Tokunaga (National Institute of Infectious Diseases, Tokyo, Japan). Flag-tagged Vpx/Vpr coding plasmids were described before in [67]. The plasmid coding HIV-1 Vif was obtained from the AIDS Reagents and Reference Program of the NIH.

The HIV-1 and SIV_MAC251_ GFP-coding lentiviral vectors and their production has been described before [67]. Briefly, these single round infectivity vectors were produced by co-transfection of HEK293T cells with 3 plasmids coding: the packaging proteins Gag-Pro-Pol and viral non structural proteins (unless otherwise specified), a mini viral genome bearing a CMV-driven eGFP reporter and the Vesicular Stomatitis Virus G envelope (VSVg) conferring ample cellular tropism to viral vectors. Virions released in the supernatant of transfected cells were then purified through a 25% sucrose cushion, resuspended and their infection titers determined onto HeLa cells or by protein content against standards of known infectivity (exo-RT activity).

Replication competent R5-tropic HIV-1 virus (Yu2) was produced by transient transfection of HEK293T cells then used to infect target cells.

miR30-shRNA hairpins were designed using the Open Biosystem Website facility and cloned in the context of an HIV-1 lentiviral construct allowing their stable expression upon viral transduction (the targeted sequences are listed in Table 1, [67]). In this case, HIV-1 vectors bearing a miR30-shRNA expression cassette were produced as mentioned above and quantified by exo-RT over standards of known infectious titer. For A3A silencing, a mixture of the 3 targeting miR30-shRNAs was used in the transfection during the step of virus production and the overall DNA ratio of packaging to vector constructs was kept constant between specific and control miR30-shRNA coding vectors.

**Table 1:** List of primers and sequences used.

Gene	Forward Primer	Reverse Primer
GFP	GAACGGCATCAAGGTGAACT	TGCTCAGGTAGTGGTTGTCG
Actin	AAGATCTGGCACCACACCTTCT	TTTTCACGGTTGGCCTTAGG

Gene	Acc. N°	miR-sh RNA target sequence
Luciferase	DQ188838	AGCTCCCGTGAATTGGAATCC
Apobec3A	nm_145699	CCCTAAGAGAATGTTGGTGAAG
Apobec3A	nm_145699	CGCTCAGTAACTGTGTCATGAA
Apobec3A	nm_145699	CGCTCAATATTCTCAGAATAAT

Gene	Acc. N°	siRNA target sequence
ctl	-	UAACGACGCGACGACGUAATT
Apobec3A	nm_145699	UGACCUACGAUGAAUUUAATT
Apobec3A	nm_145699	GUAUGCUCCCGAUCAAGUATT

### Infections

Infections were carried out for 2 hrs on 10^5^ cells prior to extensive cells washing. The percentage of infected cells was monitored 3 to 4 days post infection by flow cytometry. For miR-shRNA lentiviral vector-mediated silencing, infections were carried out on 5.10^6^ monocytes for 24 hrs prior to the addition of fresh media. Cells were then differentiated for 5 days in macrophages or DCs upon incubation with the appropriate cytokine cocktail. Cells were challenged with GFP-coding vectors four days afterwards. A multiplicity of infection (MOI) comprised between 3 and 5 was used for both silencing and challenging vectors.

Replicative infections were carried out as described above at MOI of 0,05 except that a fraction of the supernatant was stored and replaced with fresh media every 2–4 days. Viral spread through the culture was monitored by exo-RT. When used to reveal deamination of produced vDNA, virions were treated twice with 20 U/ml RNase free DNase RQ1 (Promega) for 2 hours at 37°C, and DNA extracted from infected cells was treated overnight at 37°C with DpnI. vDNA obtained after PCR amplification was cloned and sequenced.

### Transfection of siRNAs

M-SCF-differentiated macrophages were transfected with a total of 100 pmol of siRNAs (Darmacon) using Lipofectamine2000, according to the manufacturer's protocol (Invitrogen). After 6 hours, fresh media was added and cells were analyzed 48 hours post transfection by Western blot or challenged.

### Quantitative real time PCRs

RNA was extracted on silica columns (NucleoSpin RNA XS; Macherey-Nagel) and reverse transcribed after DNAse treatment, using the SuperScript II Reverse Transcriptase (Invitrogen). Quantitative PCR was performed on a StepOnePlus Real-time PCR system (Applied Biosystems) using the FastStart Universal SYBR Green Master mix (Roche Diagnostics). Primers specific for each member of the APOBEC3 family genes and house-keeping genes (TBP, HPRT1 and RPL13A) had been previously described [44]. The level of expression of these genes does not vary among the different cell types and conditions used here. Results are expressed in mRNA copies per µg of total RNA.

For the analysis of vDNA produced upon infection, cells were lyzed 24 hours post infection and DNA amplified using primers specific for the GFP sequence carried by the challenge virus (see Table 1). Control infections were carried out in the presence of RT inhibitors (Nevirapine and AZT, at 20 µg/ml, obtained through the AIDS Reagents and Reference Program of the NIH). Values were first normalized for the amount of cellular DNA (actin) then subtracted for the values obtained for each sample in control infections performed in the presence of RT inhibitors. Experiments were discarded if the latter represented values higher than 1/10 of the ones obtained in the absence of RT inhibitors.

### Co-Immunoprecipitations

PMA-differentiated THP-1 cells were lysed in SD buffer 150 mM (50 mM Tris/HCl pH 7.4, 150 mM NaCl, 0,5% Triton-X100) in presence of a cocktail of protease inhibitors (Roche). The lysate was passed through a nichel column with immobilized HIV-1 Vpr protein, then on a second column containing SIV_MAC_ Vpx. Columns were washed thrice with SD buffer 400 mM (50 mM Tris/HCl pH 7.4, 400 mM NaCl, 0,5% Triton-X100) prior to analysis by WB. Transfections of HEK293T cells were carried out in 6 well plates using a 1∶1 ratio of the indicated Vpx/Vpr and APOBEC3 proteins. Thirty-six hours post transfection, cells were lyzed in SD buffer 150 mM, then immunoprecipitated by addition of anti-Flag antibody-coated beads (Sigma) for 3 hours at +4°C. Beads were washed thrice with SD buffer 400 mM prior to WB analysis.

### DuoLink immunofluorescence

HeLa cells were seeded on coverlips and transfected with 300 ng of DNA using JetPei, according to the manufacturer's protocol (Polyplus). Twenty-four hours post transfection, cells were washed twice in PBS and fixed in 3% Paraformaldehyde in PBS (PFA). Free aldehydes were quenched by the addition of 50 mM NH_4_Cl in PBS before an additional wash in PBS. Cells were then permeabilized in 0,2% Triton-X100 for 5 min and saturated for 45 min in 0,2% gelatin from cold water skin fish (SIGMA) before immunostaining with DuoLink kit (Eurogentec), following the manufacturer's protocol. Image acquisition was carried out on an Axiovert 100 M Zeiss LSM 510 confocal microscope.

### Accession numbers

APOBEC3A: Ensembl:ENSG00000128383; APOBEC3B: Ensembl:ENSG00000179750; APOBEC3C: Ensembl:ENSG00000244509; APOBEC3D/E: Ensembl:ENSG00000243811; APOBEC3F: Ensembl:ENSG00000128394; APOBEC3G: Ensembl:ENSG00000239713; APOBEC3H: Ensembl:ENSG00000100298.

## Supporting Information

Figure S1 **IFNα increases the levels of A3A at the mRNA and protein level.** To determine the effect of IFNα on the mRNA levels of A3A, cells were incubated for 24 hrs with IFNα prior to cell lysis and analysis by RT-qPCR and WB (A and B, respectively). PBLs were incubated with PHA/IL2 in addition to IFNα or only with IFNα (activated and quiescent PBLs). The graph present data obtained from 3 donors, while the panels present a representative WB. Quiescent PBLs are shown here only at the mRNA level as a comparison to activated PBLs.
(TIF)

Figure S2 **The ApoC17 antibody recognizes specifically A3A and A3G.** To determine the specificity of the ApoC17 antibody, HEK293T cells were transfected with DNAs coding for HA-tagged versions of the different A3 family members. Cell lysates were then probed with an anti-HA or with an anti-ApoC17 antibody. As described previously, this antibody recognizes A3A and A3G, clearly distinguishable for their different size.
(TIF)

Figure S3 **Type I IFN is not secreted during spreading infection of HIV-1 in primary macrophages.** To determine the presence of secreted IFN in the supernatant of HIV-infected cultures, we used a well established method based on the extreme susceptibility of the Vesicular Stomatitis Virus (VSV) to all type α and β IFN subtypes. Briefly, the supernatant to test is incubated either directly or upon dilution with A549 to induce an antiviral state and cells are then challenged with GFP-coding VSV. The percentage of GFP-positive A549 cells is then determined 24 hours post infection. A standard curve is obtained with exogenously added IFNα2 allowing for a precise quantification of the amount of IFN secreted in a given supernatant (a typical example of standard curve is depicted in A). In our hands the lower limit of detection of IFN in this assay is of 10 U/ml, well below the concentrations of IFN used in the literature to induce an antiviral state (100 U/ml and higher). B) The graph presents the results obtained using this assay with supernatants obtained from mock and HIV-1 infected macrophages at different time points (from 4 different experiments). The supernatants obtained from plasmacytoid DCs unstimulated or incubated for 24 hours with inactivated Influenza and HIV-1 viruses were used as negative and positive controls. Given the high levels of secretion, in this case supernatants were diluted to be within the linear range of the assay.
(TIF)

Figure S4 **IFNα does not modify the susceptibility of PBLs to HIV-1 infection.** A) The effect of IFNα on the infectivity of control- or A3A-silenced PBLs was determined by incubating PHA/IL2 stimulated and stably silenced PBLs for 24 hrs with IFNα prior to infection with HIV-1. The graph presents data obtained in 3 to 4 independent experiments and with cells of different donors.
(TIF)

Figure S5 **Specificity of the A3A knockdowns.** A) To determine the specificity of the A3A knockdown, RNA was extracted from silenced macrophages and mRNA variations of all the members of the APOBEC3 family were determined by RT-qPCR. The graph presents data obtained from 4 different donors. B) In an alternative approach, A3A silencing was achieved upon siRNA-mediated transfection using siRNAs targeting sequences on the A3A mRNA that were distinct than those targeted in A. Cells were challenged with GFP-coding HIV-1 and analyzed by WB and flow cytometry 3–4 days post-infection. The graph presents data obtained with 3 donors.
(TIF)

Figure S6 **Vpx, but not A3A increases the kinetics of reverse transcription in primary macrophages.** We have previously determined that SIV_MAC_ Vpx exerts a positive effect on the infectivity of HIV-1 by speeding up reverse transcription during the infection of DCs. Here, control and A3A silenced macrophages were challenged with HIV-1 GFP vectors in comparison with infections carried out in the presence of non-infectious SIV_MAC_-derived VLPs-bearing Vpx (used as Vpx carriers). The kinetics of reverse transcription of infectious vDNA were determined as in Figure 5B. Contrarily to A3A knockdown, infections carried out in the presence of VLPs-Vpx increase the speed of reverse transcription. For direct comparison, the same representative experiment as the one depicted in Fig. 5B is presented here (out of 2).
(TIF)
