# Supplementary figures and images for: APOBEC3A Is a Specific Inhibitor of the Early Phases of HIV-1 Infection in Myeloid Cells
Source: PLoS Pathog. 2011 Sep 22;7(9):e1002221. doi: 10.1371/journal.ppat.1002221 (PMC3178557; doi:10.1371/journal.ppat.1002221)

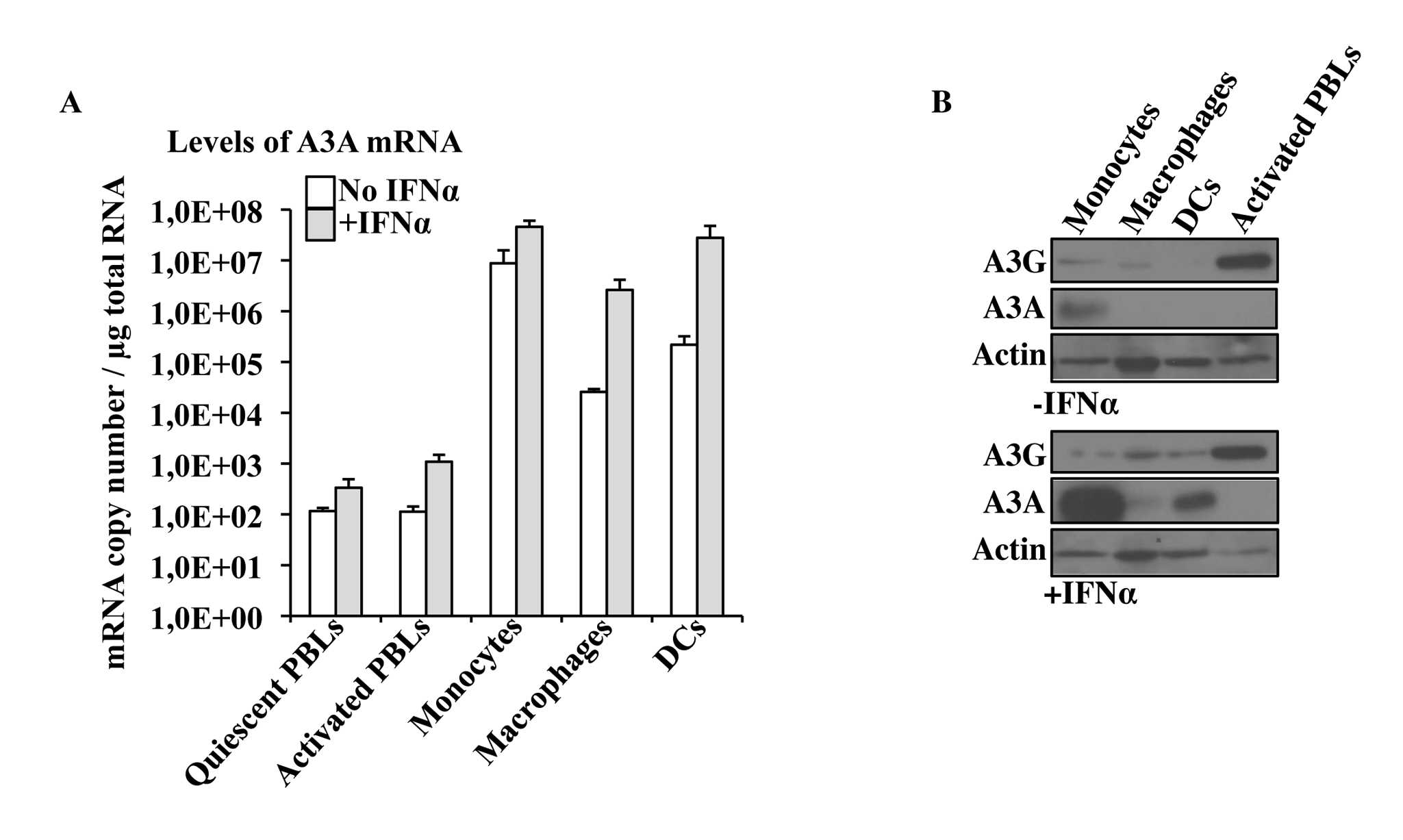

Supplement: Figure S1 — IFNα increases the levels of A3A at the mRNA and protein level. To determine the effect of IFNα on the mRNA levels of A3A, cells were incubated for 24 hrs with IFNα prior to cell lysis and analysis by RT-qPCR and WB (A and B, respectively). PBLs were incubated with PHA/IL2 in addition to IFNα or only with IFNα (activated and quiescent PBLs). The graph present data obtained from 3 donors, while the panels present a representative WB. Quiescent PBLs are shown here only at the mRNA level as a comparison to activated PBLs. (TIF) [file ppat.1002221.s001.tif]

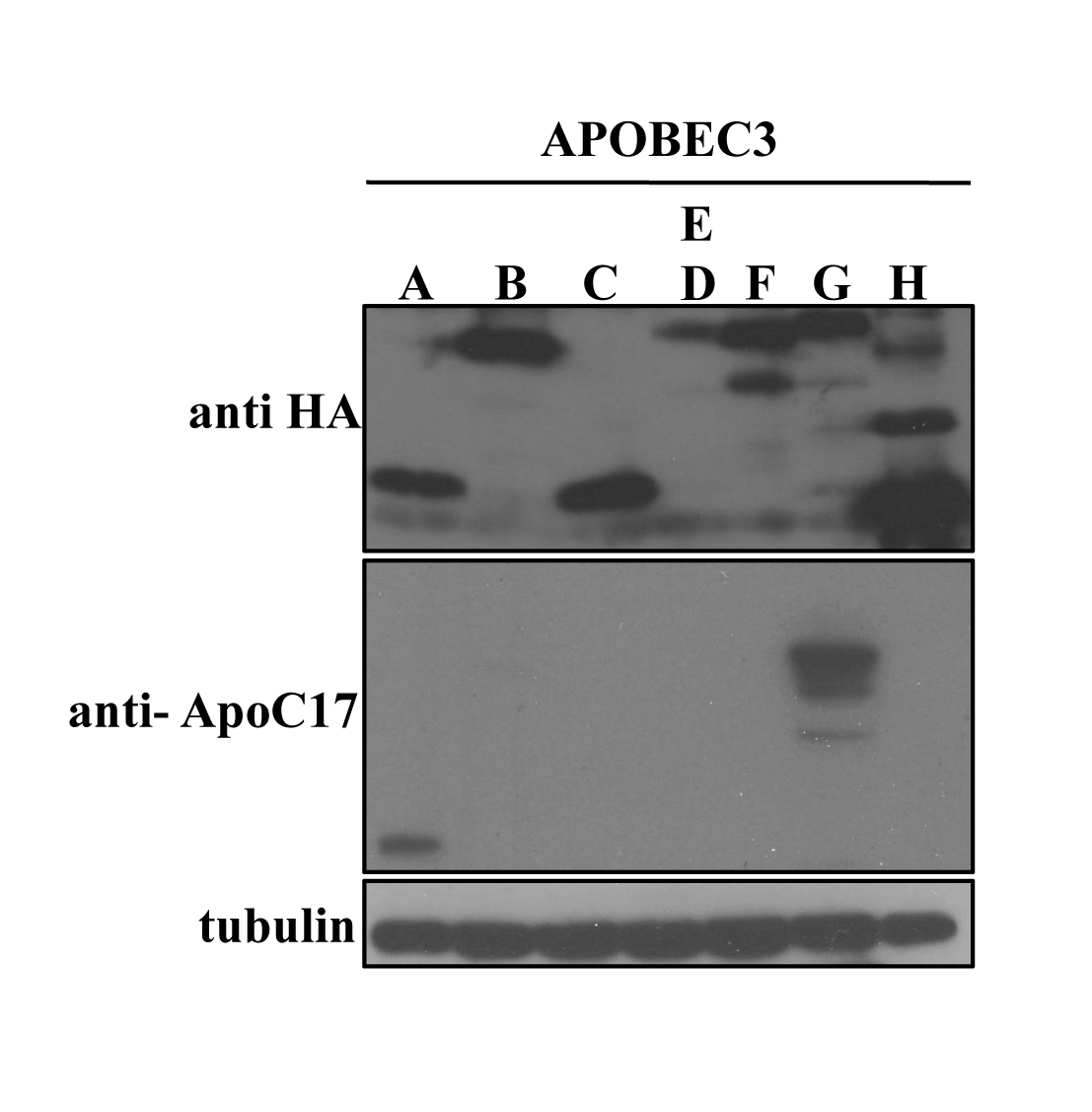

Supplement: Figure S2 — The ApoC17 antibody recognizes specifically A3A and A3G. To determine the specificity of the ApoC17 antibody, HEK293T cells were transfected with DNAs coding for HA-tagged versions of the different A3 family members. Cell lysates were then probed with an anti-HA or with an anti-ApoC17 antibody. As described previously, this antibody recognizes A3A and A3G, clearly distinguishable for their different size. (TIF) [file ppat.1002221.s002.tif]

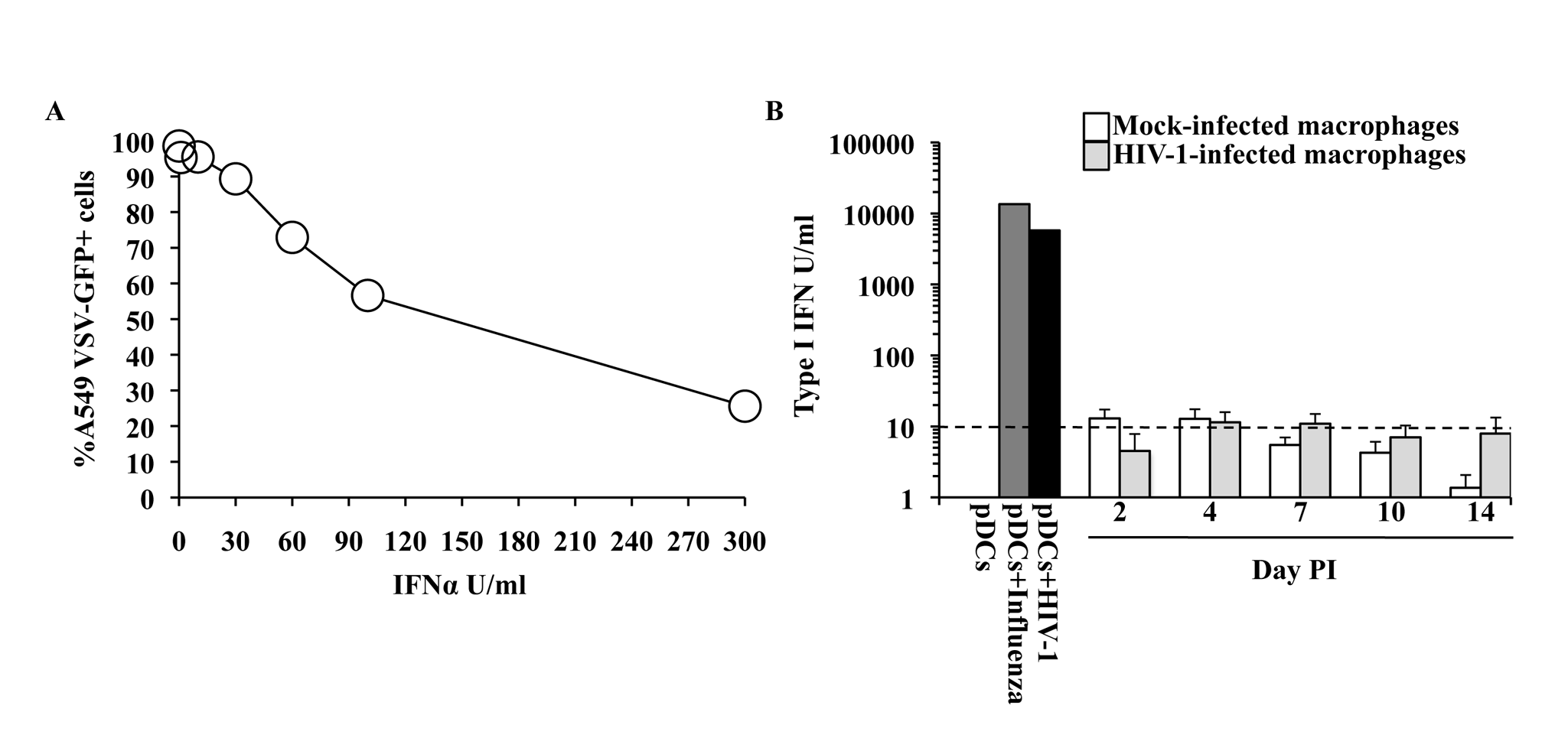

Supplement: Figure S3 — Type I IFN is not secreted during spreading infection of HIV-1 in primary macrophages. To determine the presence of secreted IFN in the supernatant of HIV-infected cultures, we used a well established method based on the extreme susceptibility of the Vesicular Stomatitis Virus (VSV) to all type α and β IFN subtypes. Briefly, the supernatant to test is incubated either directly or upon dilution with A549 to induce an antiviral state and cells are then challenged with GFP-coding VSV. The percentage of GFP-positive A549 cells is then determined 24 hours post infection. A standard curve is obtained with exogenously added IFNα2 allowing for a precise quantification of the amount of IFN secreted in a given supernatant (a typical example of standard curve is depicted in A). In our hands the lower limit of detection of IFN in this assay is of 10 U/ml, well below the concentrations of IFN used in the literature to induce an antiviral state (100 U/ml and higher). B) The graph presents the results obtained using this assay with supernatants obtained from mock and HIV-1 infected macrophages at different time points (from 4 different experiments). The supernatants obtained from plasmacytoid DCs unstimulated or incubated for 24 hours with inactivated Influenza and HIV-1 viruses were used as negative and positive controls. Given the high levels of secretion, in this case supernatants were diluted to be within the linear range of the assay. (TIF) [file ppat.1002221.s003.tif]

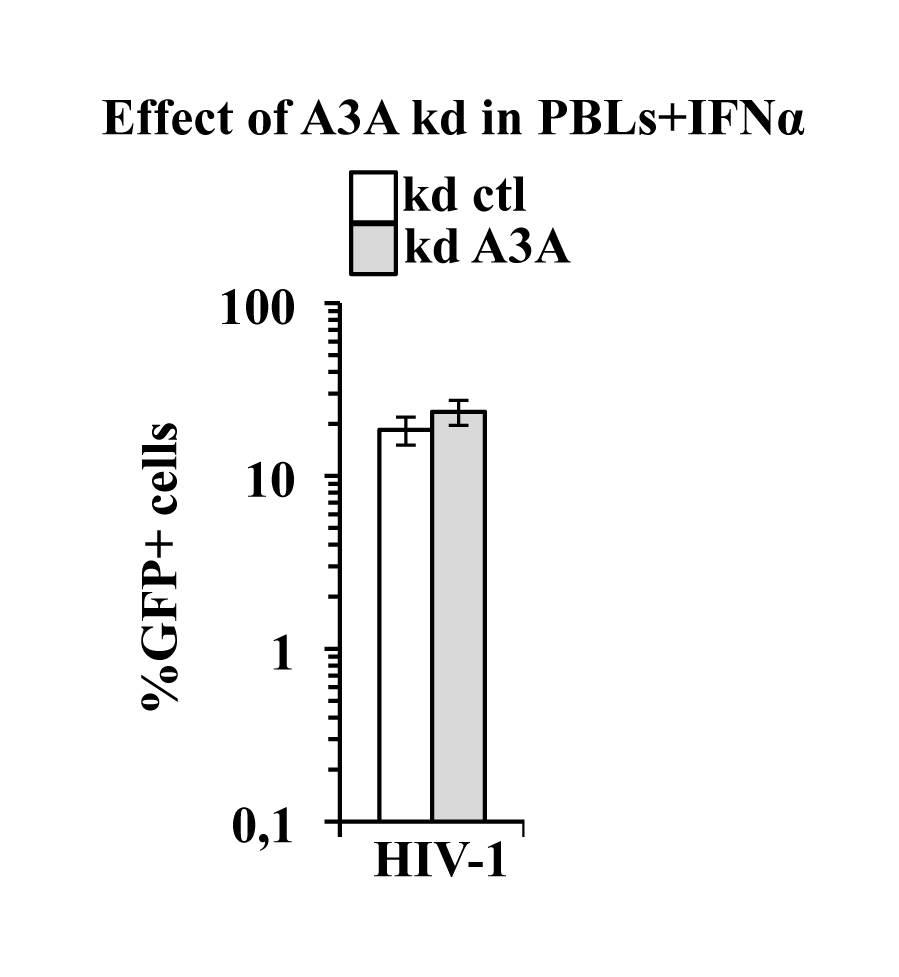

Supplement: Figure S4 — IFNα does not modify the susceptibility of PBLs to HIV-1 infection. A) The effect of IFNα on the infectivity of control- or A3A-silenced PBLs was determined by incubating PHA/IL2 stimulated and stably silenced PBLs for 24 hrs with IFNα prior to infection with HIV-1. The graph presents data obtained in 3 to 4 independent experiments and with cells of different donors. (TIF) [file ppat.1002221.s004.tif]

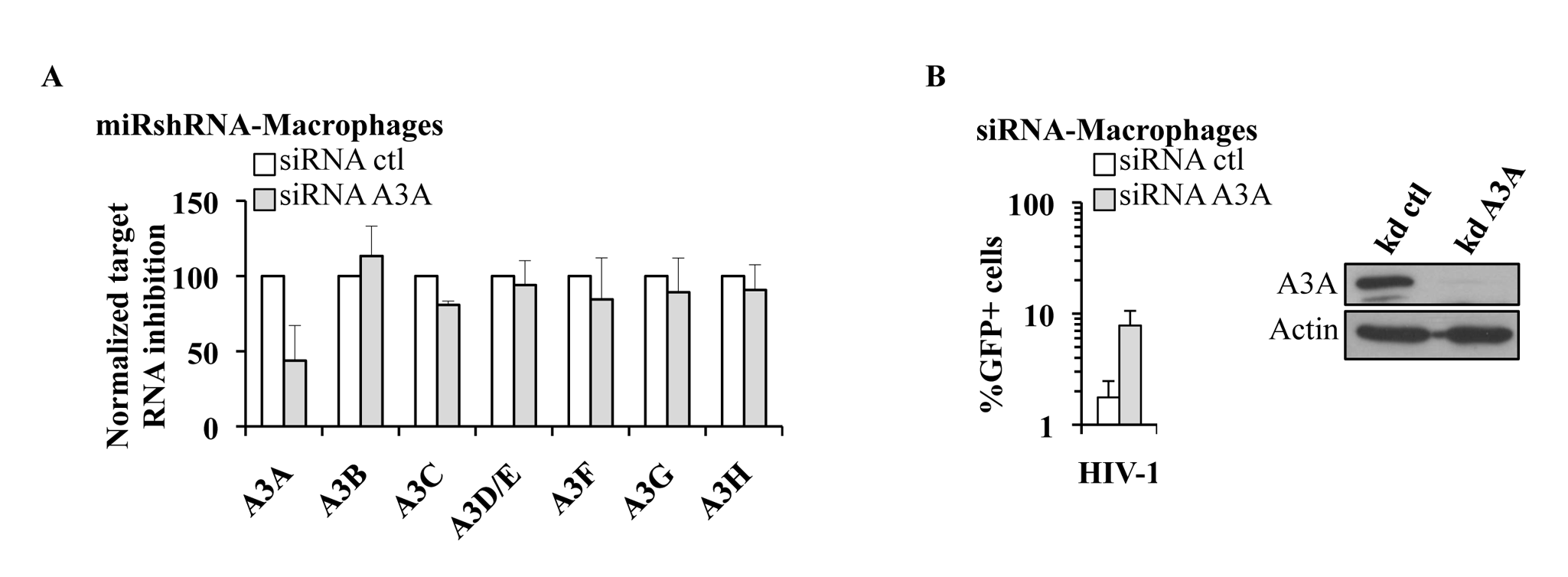

Supplement: Figure S5 — Specificity of the A3A knockdowns. A) To determine the specificity of the A3A knockdown, RNA was extracted from silenced macrophages and mRNA variations of all the members of the APOBEC3 family were determined by RT-qPCR. The graph presents data obtained from 4 different donors. B) In an alternative approach, A3A silencing was achieved upon siRNA-mediated transfection using siRNAs targeting sequences on the A3A mRNA that were distinct than those targeted in A. Cells were challenged with GFP-coding HIV-1 and analyzed by WB and flow cytometry 3–4 days post-infection. The graph presents data obtained with 3 donors. (TIF) [file ppat.1002221.s005.tif]

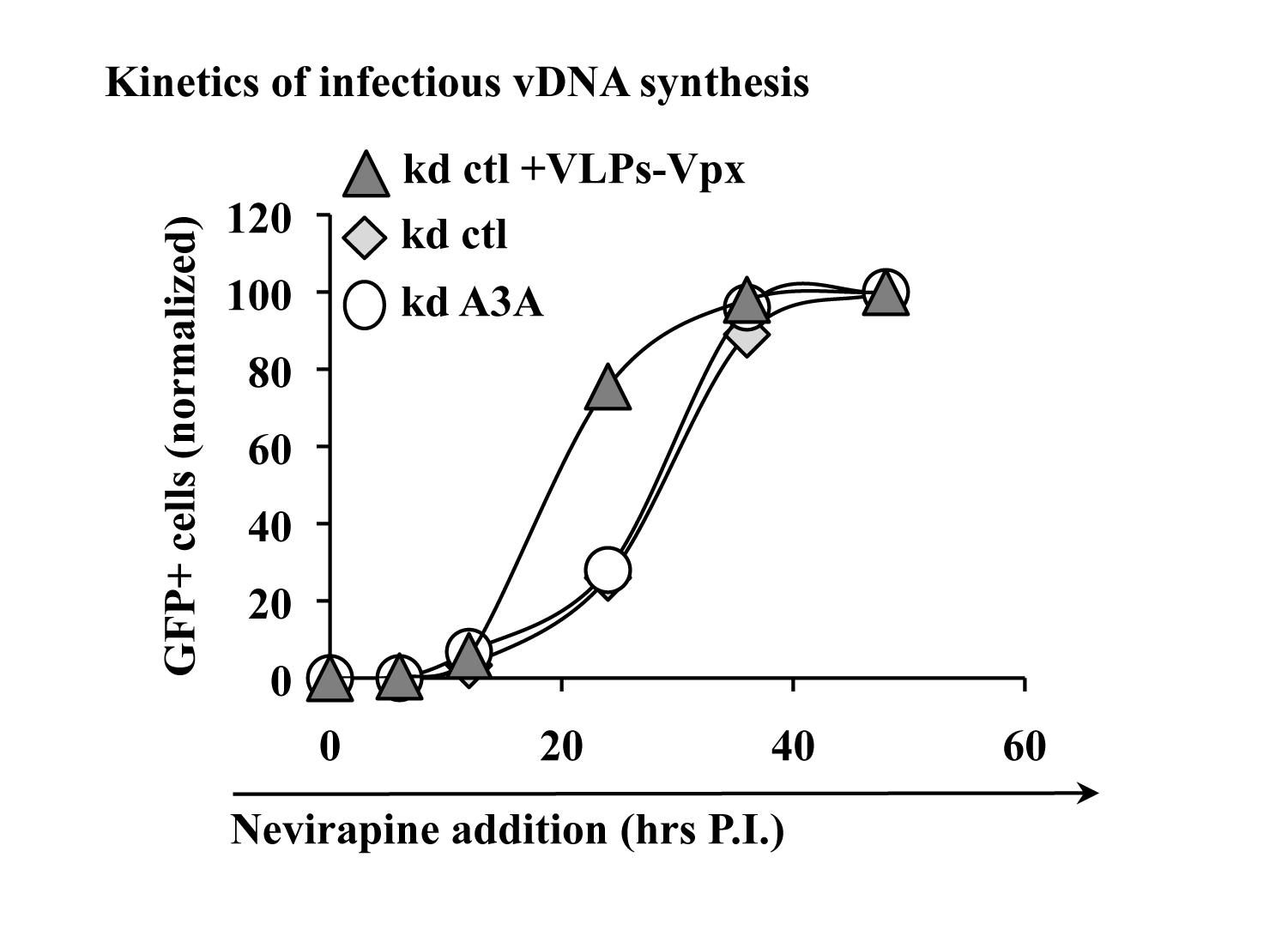

Supplement: Figure S6 — Vpx, but not A3A increases the kinetics of reverse transcription in primary macrophages. We have previously determined that SIVMAC Vpx exerts a positive effect on the infectivity of HIV-1 by speeding up reverse transcription during the infection of DCs. Here, control and A3A silenced macrophages were challenged with HIV-1 GFP vectors in comparison with infections carried out in the presence of non-infectious SIVMAC-derived VLPs-bearing Vpx (used as Vpx carriers). The kinetics of reverse transcription of infectious vDNA were determined as in Figure 5B. Contrarily to A3A knockdown, infections carried out in the presence of VLPs-Vpx increase the speed of reverse transcription. For direct comparison, the same representative experiment as the one depicted in Fig. 5B is presented here (out of 2). (TIF) [file ppat.1002221.s006.tif]
